# ROR2 induces cell apoptosis via activating IRE1α/JNK/CHOP pathway in high-grade serous ovarian carcinoma in vitro and in vivo

**DOI:** 10.1186/s12967-019-02178-x

**Published:** 2019-12-26

**Authors:** Rui Li, Tianfeng Liu, Juanjuan Shi, Wenqing Luan, Xuan Wei, Jiangtao Yu, Hongluan Mao, Peishu Liu

**Affiliations:** 1 Department of Gynecology and Obstetrics, Qilu Hospital of Shandong University, 107 Wenhua Xi Road, Jinan, 250012 Shandong People’s Republic of China; 2 Department of Gynecology and Obstetrics, Linyi People’s Hospital, 27 Jiefang Road, Linyi, 276003 Shandong People’s Republic of China; 3 Department of Gynecology and Obstetrics, Affiliated Tengzhou Center People’s Hospital of Jining Medical University, 181 Xing Tan Road, Tengzhou, 277599 Shandong People’s Republic of China

**Keywords:** ROR2, High-grade serous ovarian carcinoma, Apoptosis, IRE1α/JNK/CHOP pathway

## Abstract

**Background:**

Epithelial ovarian cancer (EOC) is the most lethal cancer in female genital tumors. New disease markers and novel therapeutic strategies are urgent to identify considering the current status of treatment. Receptor tyrosine kinases family plays critical roles in embryo development and disease progression. However, ambivalent research conclusions of ROR2 make its role in tumor confused and the underlying mechanism is far from being understood. In this study, we sought to clarify the effects of ROR2 on high-grade serous ovarian carcinoma (HGSOC) cells and reveal the mechanism.

**Methods:**

Immunohistochemistry assay and western-blot assay were used to detect proteins expression. ROR2 overexpression adenovirus and Lentivirus were used to create ROR2 overexpression model in vitro and in vivo, respectively. MTT assay, colony formation assay and transwell assay were used to measure the proliferation, invasion and migration ability of cancer cells. Flow cytometry assay was used to detect cell apoptosis rate. Whole transcriptome analysis was used to explore the differentially expressed genes between ROR2 overexpression group and negative control group. SiRNA targeted IRE1α was used to knockdown IRE1α. Kira6 was used to inhibit phosphorylation of IRE1α.

**Results:**

Expression of ROR2 was significantly lower in HGSOC tissues compared to normal fallopian tube epithelium or ovarian surface epithelium tissues. In HGSOC cohort, patients with advanced stages or positive lymph nodes were prone to express lower ROR2. Overexpression of ROR2 could repress the proliferation of HGSOC cells and induce cell apoptosis. RNA sequencing analysis indicated that ROR2 overexpression could induce unfold protein response. The results were also confirmed by upregulation of BIP and phosphorylated IRE1α. Furthermore, pro-death factors like CHOP, phosphorylated JNK and phosphorylated c-Jun were also upregulated. IRE1α knockdown or Kira6 treatment could reverse the apoptosis induced by ROR2 overexpression. Finally, tumor xenograft experiment showed ROR2 overexpression could significantly repress the growth rate and volume of transplanted tumors.

**Conclusions:**

Taken together, ROR2 downregulation was associated with HGSOC development and progression. ROR2 overexpression could repress cell proliferation and induce cell apoptosis in HGSOC cells. And the underlying mechanism might be the activation of IRE1α/JNK/CHOP pathway induced by ROR2.

## Introduction

Epithelial ovarian cancer (EOC) is the most lethal cancer in female genital tumors due to absence of screening methods and limited treatments to recurrence [1]. Among the main subtypes of this disease, high-grade serous ovarian carcinoma (HGSOC) accounts for majority part [2]. Prognosis of patients with advanced stages (International Federation of Gynecology and Obstetrics, FIGO stage III–IV) has met marginal improvement even with unceasing development of technology and medicine [1]. Hence, it’s urgent to identify new disease markers and discover novel therapeutic strategies.

Receptor tyrosine kinases (RTKs) family are found to play critical roles in embryo development and disease progression [3]. Receptor tyrosine kinase-like orphan receptors (RORs) form a small subfamily of RTKs, featured by a conserved domain architecture and composed of two members, ROR1 and ROR2, located in chromosome 1 and 9, respectively [4, 5]. ROR RTKs have always been deemed to be tumor promoters, especially ROR1, which has been reported to be involved in tumor progression in multiple cancers [6–10]. Different from ROR1, character of ROR2 in tumors has faced a bit of controversy recently. Compared with the opinion that ROR2 works as tumor promoter in initial studies, more and more researches show ROR2 plays dual roles in tumors depending on tumor type and tumor context. ROR2 played as tumor promoter in renal cancer [11], chronic lymphocytic leukemia [12], malignant melanoma [13], whereas its high expression was shown to be related with better prognosis in patients with colorectal cancer [14, 15], liver cancer [16], medulloblastoma [17] and endometrial cancer [18]. Different roles were even played within the same kind of tumor [19]. The research status urged us to review ROR2 considering cell type and context. ROR RTKs were referred as orphan receptors for decades initially as no ligands were identified [20]. Then intimate connection with Wnt family indicates that ROR2 regulates tumor biological behavior through canonical or non-canonical Wnt signaling pathway [20–22], and additional downstream effectors include Rho-family GTPases, JNK cascade, and the like [23–25]. ROR2 has also been found to antagonize AKT signaling, thus inhibited the growth, invasion and migration of tumor cells [26]. For ROR2 and its downstream cascades there is a need for more research.

In this study, we detected expression of ROR2 was significantly lower in HGSOC tissues compared to normal fallopian tube epithelium (FTE) or ovarian surface epithelium (OSE) tissues. HGSOC patients with advanced FIGO stages or positive lymph nodes were prone to express lower ROR2. Data showed patients with advanced stages expressed lower ROR2. We further demonstrated ROR2 inhibited cell proliferation and induced cell apoptosis in HGSOC cells. RNA-sequencing and western-blot results confirmed ROR2 induced endoplasmic reticulum stress (ERS), which clarified the underlying mechanism. All we found revealed a novel character ROR2 played in HGSOC.

## Materials and methods

### Cell lines and culture conditions

The human epithelial ovarian cancer cell lines HEY, OV-90 and HO-8910 were purchased from the Cell Bank of the Chinese Academy of Sciences in Shanghai, China. The HEY cells and A2780 cells were cultured in DMEM medium and RPMI-1640 medium respectively with 10% fetal bovine serum, 100 U/ml penicillin and 100 μg/ml streptomycin. The HO-8910 cells were cultured exactly like A2780 cells. The OV-90 cells were cultured in half MCDB 105 medium and half 199 medium with 15% bovine serum, 100 U/ml penicillin and 100 μg/ml streptomycin. Cells were cultured at 37 °C in 5% CO_2_ and 95% air.

### Chemical inhibitors and antibodies

The chemical Kira6 (IRE1α kinase inhibitor) was purchased from Selleck, TX, USA. The antibodies ROR2, caspase3, caspase7, PARP, cleaved caspase3, cleaved caspase7, cleaved PARP, BIP, CHOP, IRE1α, c-Jun, phosphorylated IRE1α, phosphorylated JNK(Thr183/Tyr185), phosphorylated c-Jun(Ser73) and β-actin used for western-blot assay were purchased from Cell Signaling Technology, MA, USA. The antibodies Bcl-2, Bax and JNK used for western blot assay were purchased from Santa Cruz, CA, USA. The antibody phosphorylated IRE1α(Ser724) used for western-blot assay was purchased from Abcam, Cambridge, UK. The antibodies ROR2, BIP and CHOP used for immunohistochemistry were purchased from Abcam, Cambridge, UK.

### Tissue samples for western-blot assay

Tissue samples were collected from 23 HGSOC patients who underwent surgical resection at Qilu Hospital of Shandong University in first half of 2019. The ages ranged from 41 to 80 (median: 62 years). 21.8% patients were diagnosed at early stages (FIGO stage I–II) and the others were diagnosed at advanced stages (FIGO stage III–IV). All patients were diagnosed based on clinical protocols without previous neo-adjuvant chemotherapy or immunotherapy. Meanwhile, fimbriae of the fallopian tubes were collected from 23 patients receiving bilateral salpingectomy with benign neoplasms at the same hospital as normal control tissues.

### GEO data sets analysis

Gene expression profiles of GSE69428, GSE40595 and GSE18520 were downloaded from Gene Expression Omnibus (GEO). Dataset GSE69428 included HGSOC and paired normal FTE samples from 10 independent patients. Dataset GSE40595 included epithelial tumor samples from 32 HGSOC patients and 6 normal OSE samples. Dataset GSE18520 included 53 HGSOC tissue samples and 10 normal OSE samples. All the samples were isolated laser based microdissection using the Affymetrix human genome U133 Plus 2.0 microarray. The “limma” package was used to analyze differentially expressed genes (DEGs) between HGSOC and normal FTE or OSE samples. The adjusted *P* < 0.05 and |log2fold change (FC)| > 1 were set as the cut-off criteria. Adjusted value (adj. *P*) was applied to correct false-positives.

### Adenovirus transfection and chemical treatment

The adenovirus was purchased from Vigene Bioscience in Jinan, China. Cells were suspended with normal culture medium and seeded in 6-well plate at 20 × 10^4^ cells/well overnight to adhere. Volume of adenovirus was measured according to the MOI (volume = [cell number × MOI]/virus titer, MOI_HEY_ = 50, MOI_OV-90_ = 50, MOI_HO-8910_ = 30). After transfected in opti-MEM with adenovirus for 6 h, normal culture medium was replaced for the following cultural. To examine the effects of IRE1α kinase inhibitor on apoptosis and relevant molecules, HEY and HO-8910 cells were first infected with ROR2 overexpression or negative control adenovirus for 6 h and then treated with 2 μM Kira6 for 72 h before collecting for the following assays.

### Immunohistochemistry assay

The patient tissue chip was purchased from Alenabio, Xian, China. Fresh tissues from tumor xenograft were fixed with 4% paraformaldehyde for 24 h before dehydrated and embedded in paraffin. Tissue sections were torrefied for 1 h and dewaxed with xylene and ethyl alcohol. The microwave antigen retrieval technique was used to repair the antigen. Sections were incubated with 3% H_2_O_2_ for 20 min to block endogenous peroxidase activity and incubated with goat serum for 30 min to block non-specific antigens. Then sections were incubated with primary antibodies (ROR2, 1:400, BIP, 1:300, CHOP: 1:300) overnight at 4 °C. After incubated with biotin-labeled goat anti-rabbit IgG polymer and horseradish enzyme-labeled streptomycin for 30 min, respectively, positive signals were detected with DAB reagent and quantified by Image-Pro Plus software 6.0 (Media Cybernetics, USA). The same setting was used for all the analyzed tissues to be accurate for the staining reading. Integrated optical density (IOD) and size of the total area was measured in each field, and staining score was formulated as IOD/size.

### Western-blot assay

Cells were washed with 1× PBS for 3 times and cells lysates were prepared in RIPA lysis buffer with 1% phenylmethanesulfonyl fluoride (PMSF) and 1% sodium fluoride. Protein concentrations were detected with BCA protein assay kit (Beyotime, Beijing, China). Total proteins were separated on a 12% polyacrylamide gel and transferred to a polyvinylidene fluoride membrane. Membranes were blocked with 5% defatted milk for 1 h at room temperature and incubated with the primary antibodies overnight at 4 °C. Then membranes were washed with 1× TBS and incubated with appropriate secondary antibodies. Bands were detected using chemiluminescent substrate (Thermo Fisher Scientific Inc., MA, USA) and quantified by ImageJ software (National Institutes of Health, USA). The β-actin band was served as control.

### Quantitative real-time transcription-polymerase chain reaction

Total RNA of cells was extracted and concentration and purity were detected using spectrophotometer (Thermo Fisher Scientific Inc., MA, USA). Then the RNA (3000 ng/20 μl reaction system) was transcribed into cDNA. PCR reaction was performed on StepOne™ PCR amplifier (Applied Biosystems, USA) with SYBR-green (TAKARA, Japan) in a 10 μl reaction system, and β-actin was used as the control. Primers for human ROR2 gene were as follows: forward: 5′-GTGCGGTGGCTAAAGAATGAT-3′, reverse: 5′-ATTCGCAGTCGTGAACCATATT-3′. Relative gene expression levels were normalized to β-actin. Primers for β-actin gene were as follows: forward: 5′-CTCACCATGGATGATGATATCGC-3′, reverse: 5′-AGGAATCCTTCTGACCCATGC-3′.

### MTT assay

After transfected with negative control or ROR2 adenovirus, cells were suspended at respective concentrations (HEY 1500 cells/100 μl, OV-90 2500 cells/100 μl, HO-8910 2000 cells/100 μl) and seeded in 96-well plates (100 μl/well) overnight to adhere. 10 μl MTT solution (5 mg/ml) was added into every well at fixed time from Day1 to Day6. After incubated at 37 °C for 4 h, supernatant liquor was discarded and 100 μl DMSO was added in every well to dissolve the formazan. Absorbance was read at 490 nm using a microplate reader (Tecan Group Ltd., Männedorf, Switzerland).

### Colony formation assay

After transfected with negative control or ROR2 adenovirus for 24 h, cells were suspended at respective concentrations (HEY 500 cells/2 ml, OV-90 1000 cells/2 ml, HO-8910 500 cells/2 ml) and seeded in 6-well plate (2 ml/well). After incubated at 37 °C for 12 days, cells were fixed with 4% paraformaldehyde for 5 min and stained with crystal violet (Beyotime, Beijing, China) for 30 min.

### Transwell assay

Cells transfected with negative control or ROR2 adenovirus for 72 h were suspended at respective concentrations (HEY 5 × 10^4^ cells/200 μl for invasion and 3 × 10^4^ cells/200 μl for migration, HO-8910 8 × 10^4^ cells/200 μl for invasion and 5 × 10^4^ cells/200 μl for migration) and seeded in transwell chambers (8 μm pore size; Corning Costar, MA, USA) with or without Matrigel (60 μl, 1:9 dilution in serum free medium, BD Biosciences, CA, USA). After incubated at 37 °C for 24 h, cells were fixed with 4% paraformaldehyde for 5 min and stained with crystal violet (Beyotime, Beijing, China) for 30 min. Images were taken with JEM-1200 EX II Electron Microscope (JEOL, Tokyo, Japan).

### Flow cytometry assay

Flow cytometry assay was used to detect the apoptotic ratio of HGSOC cells. After transfected with negative control or ROR2 adenovirus for 72 h, cells were digested with tyrisin without EDTA and suspended at 1 × 10^6^ cells/ml. Cells (100 μl/tube) were dyed with 5 μl fluorescein isothiocyanate (FITC) Annexin-V and PI and 5 μl propidium iodide (PI) (BD, NJ, USA). After incubated at room temperature for 15 min, 400 μl binding buffer was added into each tube and cells were collected with flow cytometry (BD Biosciences, San Jose, CA, USA). Quantitative analysis of apoptotic ratio was performed by CellQuest Pro software (BD Biosciences, Franklin Lakes, NJ, USA).

### RNA sequencing assay

HO-8910 cells were seeded in culture dish at 1.5 × 10^6^ cells/dish (55 cm^2^) overnight to adhere. Volume of ROR2 overexpression and negative control adenovirus were calculated with MOI of 30. After transfected for 6 h, cells were changed with normal medium. After 48 h, cells were disrupted with Trizol Reagent (Thermo Fisher Scientific Inc., MA, USA) and sent to Novogene Corporation (Beijing, China) for whole transcriptome sequencing. The libraries were sequenced using the IlluminaHiSeq™ 4000 sequencing platform.

### Plasmid extraction, siRNA and transfection assay

The plasmid was purchased from Genechem, Shanghai, China. 1 μg plasmid was transfected into competent cells. Shaking flask culture with 100 μg/ml ampicillin was used for amplification of bacteria. Then plasmids were extracted with plasmid extraction kit (Omega, GA, USA) and the concentration and purity were measured with spectrophotometer (Thermo Fisher Scientific Inc., MA, USA). Lentivirus expressing ROR2 packaged with psPAX2 (Addgene, MA, USA) and pMD2G (Addgene, MA, USA) were produced in HEK293T cells with Lipofectamine 2000 (Invitrogen, CA, USA). Stable cells were selected for 10 days in medium with 4 μg/ml puromycin (Solarbio, Beijing, China) after transfection by Lentivirus for 12 h. The small interfering RNA (siRNA) targeting IRE1α was synthesized by BioSune (BioSune, Shanghai, China). Cell transfection was performed using Lipofectamine 2000 (Invitrogen, CA, USA).

### Tumor xenograft experiment

To verify the effects of ROR2 on ovarian cancer in vivo, ROR2 stable overexpression cells were constructed with Lentivirus PCMV-ROR2. Cells transfected with PCMV-NC were used as control. 1 × 10^7^ cells in 100 μl PBS were injected subcutaneously into either side of the armpit of the same 4-week-old nude female mice. Tumor sizes were measured every other day 10 days after injection. Sizes of tumors were measured (Volume = [length × width^2^]/2) with vernier caliper every other day. Mice were sacrificed 33 days after injection and tumors were removed and weighted. Part of the tumors were fixed with 4% paraformaldehyde for immunohistochemistry and the others were used for western-blot assay. The research was approved by the Experimental Animal Ethics Committee of Qilu Hospital of Shandong University (Approval number: KYLL-2016-338).

### Statistical analysis

All experiments were repeated three times at least. Data were analyzed with Mann–Whitney test, Student’s *t* test or 2way Analysis of Variance (ANOVA) test and shown as mean ± standard error of mean (SEM). Statistical significance was defined as *P* < 0.05. All the statistical analysis was performed using GraphPad Prism Version7.00 (GraphPad Software, USA).

## Results

### ROR2 was downregulated in HGSOC tissues and its expression was correlated with FIGO stages

**Fig. 1:**
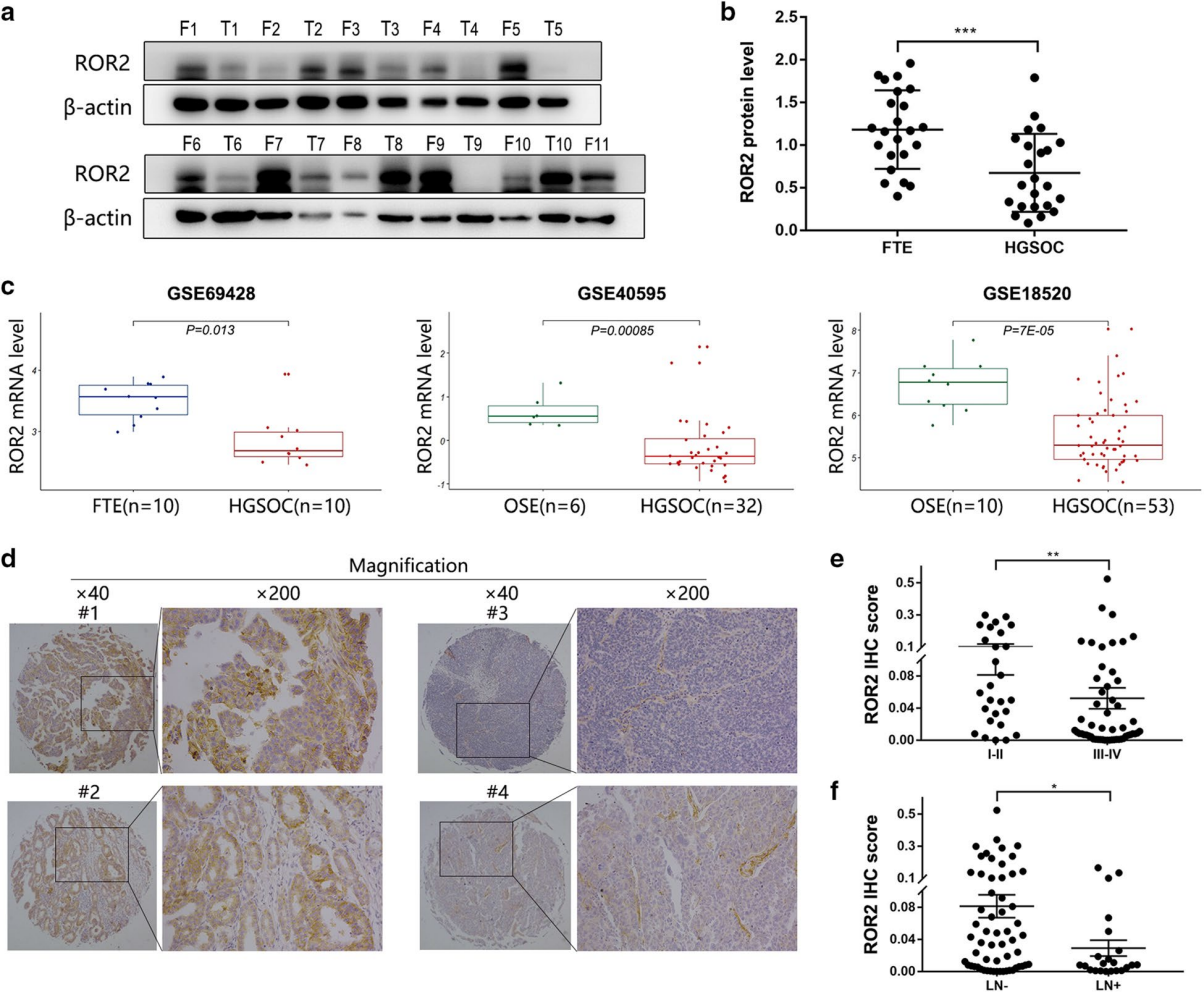
ROR2 was downregulated in HGSOC tissues and its expression was correlated with HGSOC FIGO stages. **a** Western-blot assay was used to detect the expression of ROR2 in 23 HGSOC tissues and 23 normal fallopian tube tissues. **b** Quantitation of western-blot assay bands shown in **a** using Image J. Statistical analysis was performed using Student’s *t* test. **c** Validation of ROR2 expression from 3 GEO databases, GSE69428, GSE40595 and GSE18520. The mRNA level of ROR2 was analyzed with the “limma” package using Student’s *t* test. **d** Immunohistochemical staining was used to detect the expression of ROR2 in 81 HGSOC tissue samples. **e** The intensities of IHC staining were quantitated by Image-Pro Plus 6.0. ROR2 IHC scores in different FIGO stages were analyzed with Mann–Whitney test. **f** The intensities of IHC staining were quantitated by Image-Pro Plus 6.0. ROR2 IHC scores in patients with different lymph nodes status were analyzed with Mann–Whitney test. LN−: patients with negative lymph nodes. LN+: patients with positive lymph nodes. *P < 0.05, **P < 0.01, and ***P < 0.001 for statistical analysis of the indicated groups

To detect whether ROR2 expression altered in HGSOC development, we analyzed ROR2 protein level in 23 HGSOC patients and 23 normal control tissues. The expression of ROR2 was significantly down-regulated in HGSOC tissues compared to fallopian tube tissues (Fig. 1a, b). Then gene expression data including HGSOC tissues and normal FTE or OSE tissue samples were downloaded from GEO and mRNA level of ROR2 was analyzed with the “limma” package. Consistent with our western-blot result, transcription level of ROR2 in HGSOC was lower than that in normal FTE or OSE tissues (Fig. 1c). To explore the alteration of ROR2 in HGSOC progression, we detected the expression of ROR2 by immunohistochemistry (IHC) in 81 patients with HGSOC. The data showed patients with advanced stages (FIGO stage III–IV) or positive lymph nodes were prone to express lower ROR2 (Fig. 1d–f). The clinicalpathological characteristics of HGSOC patients were shown in Tables 1, 2.

**Table 1:** Patients characteristics of western-blot assay

Characteristics	Cohort (n = 23) No. of patients (%)
Age	
< 50	4 (17.4%)
≥ 50	19 (82.6%)
Histology type	
Serous	23 (100%)
FIGO stage	
I	2 (8.7%)
II	3 (13.1%)
III	17 (73.9%)
IV	1 (4.3%)
Pathological grade	
High-grade	23 (100%)

**Table 2:** Patients characteristics of IHC assay

Characteristics	Cohort (n = 81) No. of patients (%)
Age	
< 50	31 (38.3%)
≥ 50	50 (61.7%)
Histology type	
Serous	81 (100%)
FIGO stage	
I	27 (33.3%)
II	15 (18.5%)
III	37 (45.7%)
IV	2 (2.5%)
Lymph nodes	
Negative	59 (72.8%)
Positive	22 (27.2%)
Pathological grade	
High-grade	81 (100%)

### ROR2 overexpression inhibited proliferation and colony formation of HGSOC cells

**Fig. 2:**
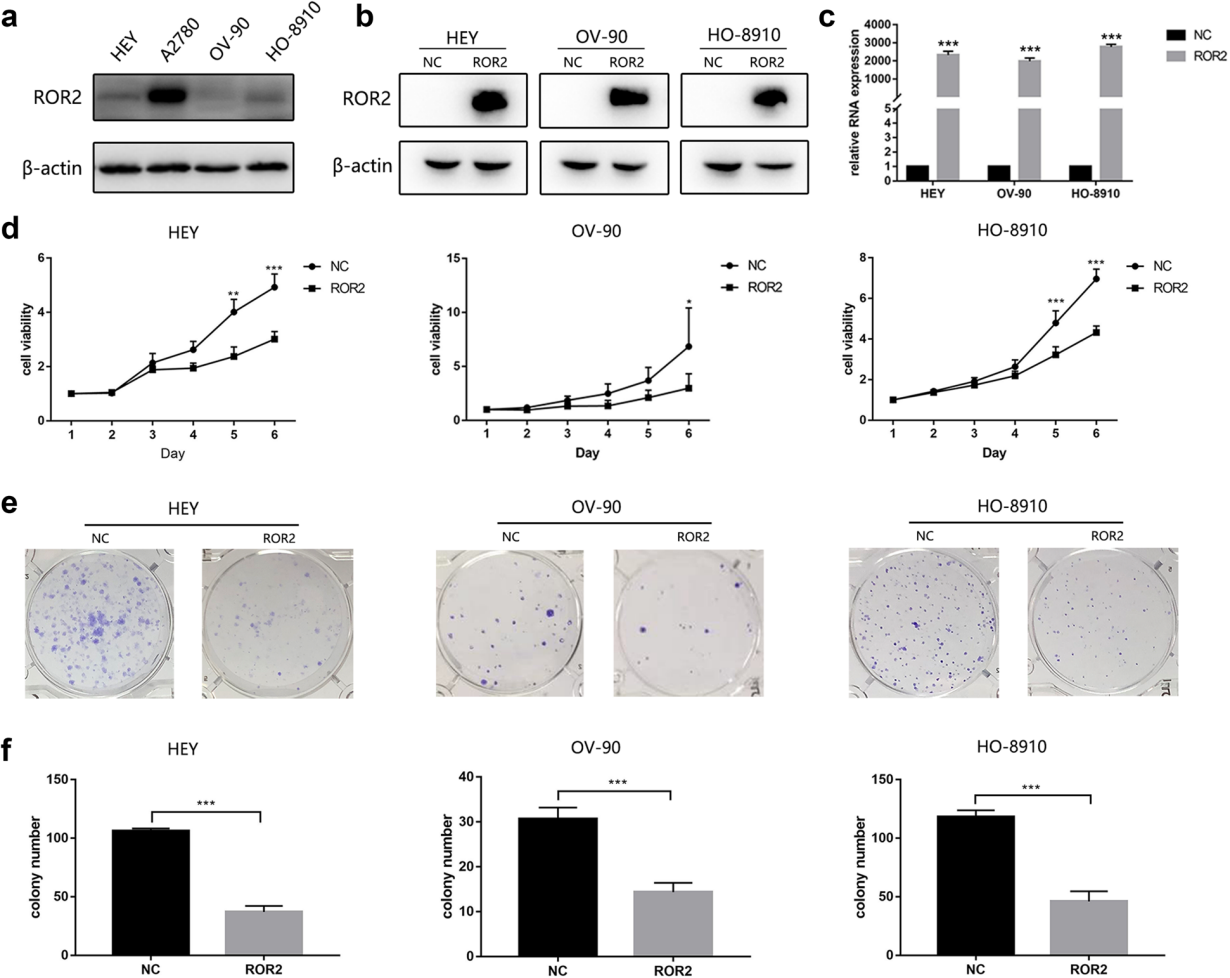
ROR2 overexpression inhibited proliferation and colony formation of HGSCO cells. **a** Western-blot assay was used to detect the expression of ROR2 in HEY, A2780, OV-90 and HO-8910 cells. **b** Western-blot assay was used to detect the protein expression of ROR2 in HEY, OV-90 and HO-8910 cells after being transfected with ROR2 overexpression or negative control adenovirus for 72 h. **c** PCR assay was used to detect the mRNA expression of ROR2 in HEY, OV-90 and HO-8910 cells after transfected with ROR2 overexpression or negative control adenovirus for 48 h. **d** HEY, OV-90 and HO-8910 cells were transfected with ROR2 overexpression or negative control adenovirus, and proliferation ability of the transfected HGSOC cells was detected at a fixed time for 6 days by MTT assay. The growth curves were analyzed using 2way ANOVA test. **e** HEY, OV-90 and HO-8910 cells were transfected with ROR2 overexpression or negative control adenovirus for 24 h before being planted into six-well plates for colony formation assay. The colony formations were harvested after 12 days. **f** Colony formations were counted and analyzed with Student’s *t* test. *NC* negative control. All experiments were repeated three times at least. *P < 0.05, **P < 0.01 and ***P < 0.001 for statistical analysis of the indicated groups

Then we detected the expression of ROR2 in 4 HGSOC cell lines. The result showed that ROR2 was barely expressed in HEY, OV-90 and HO-8910 cells compared to A2780 cells (Fig. 2a). Thus, we chose these three cell lines to create ROR2 overexpression model. Western-blot and RT-PCR results confirmed successful overexpression of ROR2 (Fig. 2b, c). After successful overexpression of ROR2 in HEY, OV-90 and HO-8910 cells, MTT result showed that the proliferation ability was significantly repressed (Fig. 2d). Then we used colony formation assay to measure the long-term effects of ROR2 on the proliferation ability of HGSOC cells. The result was consistent with the MTT assay (Fig. 2e, f). The transwell assay showed the invasion and migration abilities of HEY and HO-8910 cells in ROR2 overexpression group were significantly suppressed compared to the negative control (NC) group (Additional file 1: Figure S1a, b). Then we detected the expression of epithelial mesenchymal transition (EMT) related markers, like N-cadherin, Vimentin and Keratin to verify the result. However, the expression of these proteins did not change significantly (Additional file 1: Figure S1c).

### ROR2 overexpression induced cell apoptosis of HGSOC cells

**Fig. 3:**
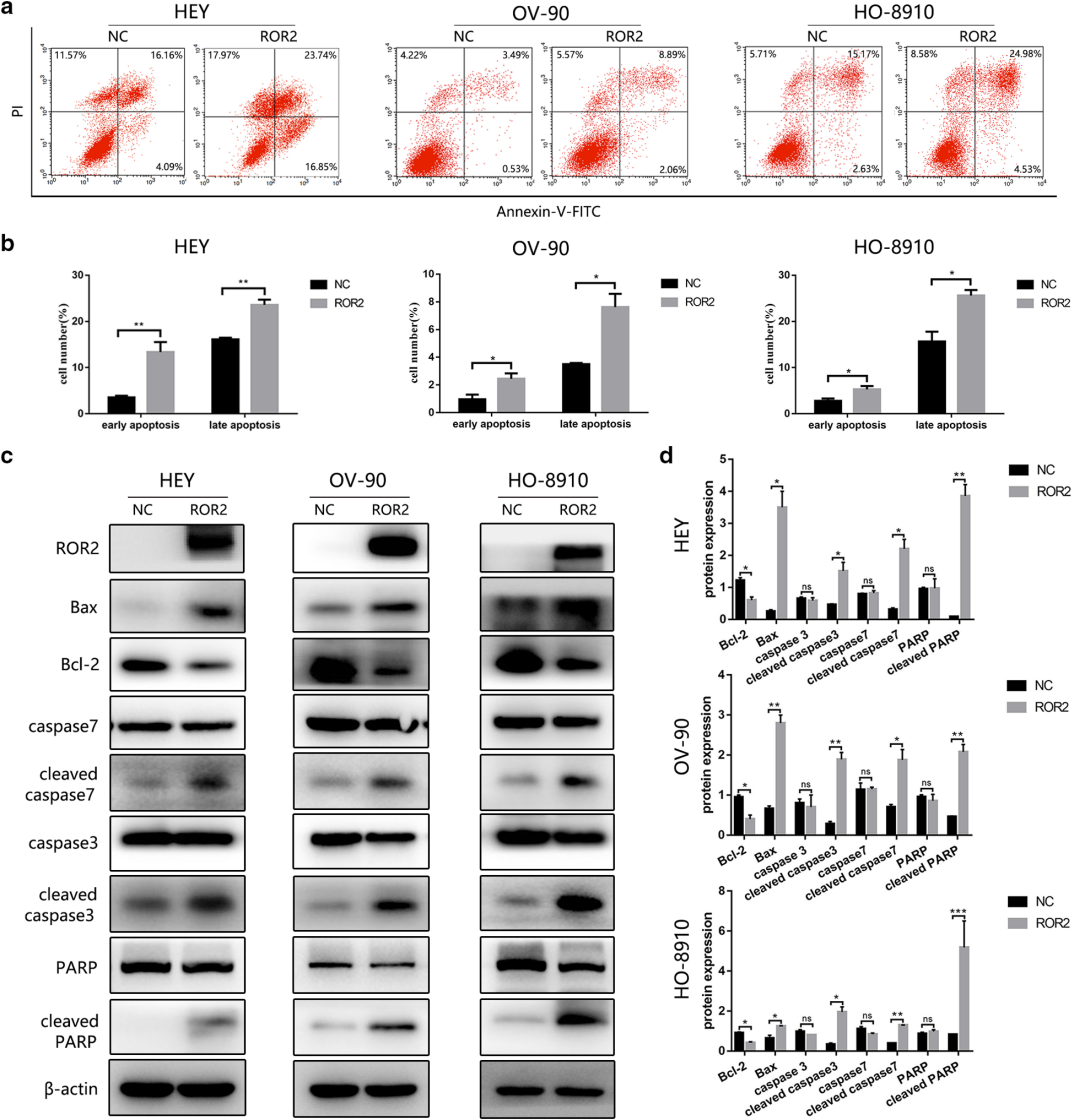
ROR2 overexpression induced cell apoptosis of HGSCO cells. **a** HEY, OV-90 and HO-8910 cells were transfected with ROR2 overexpression or negative control adenovirus for 72 h. Apoptosis was detected by flow cytometry after staining with FITC Annexin-V and PI. **b** Quantitive analysis of apoptotic ratio in HEY, OV-90 and HO-8910 cells with CellQuest Pro software. Statistical analysis was performed with GraphPad Prism using Student’s *t* test. **c** HEY, OV-90 and HO-8910 cells were transfected with ROR2 overexpression or negative control adenovirus for 72 h. Expression of ROR2, Bcl-2, Bax, caspase3, cleaved caspase3, caspase7, cleaved caspase7, PARP and cleaved PARP were determined by western-blot assay. β‑actin was used as a loading control. **d** Quantitation of wester-blot assay bands shown in **c** using Image J. Statistical analysis was performed using Student’s *t* test. NC: negative control. All experiments were repeated three times at least. *P < 0.05, **P < 0.01 and ***P < 0.001 for statistical analysis of the indicated groups

Considering that the decrease of cell viability could result from the induction of cell apoptosis, flow cytometry assay was used to detect whether upregulation of ROR2 could induce apoptosis of EOC cells. After transfected with ROR2 overexpression adenovirus for 72 h, HEY, OV-90 and HO-8910 cells went significantly apoptosis indicated by positive staining with Annexin-V and PI compared to the NC group (Fig. 3a, b). Then western-blot assay were used to detect the expression of proteins involved in apoptosis. Consistent with flow cytometry analysis, Bcl-2 was significantly downregulated, while Bax, cleaved caspase7, cleaved caspase3 and cleaved PARP were significantly upregulated in ROR2 overexpression group (Fig. 3c, d). These results suggested that upregulation of ROR2 could induce cell apoptosis in ovarian cancer cells.

### ROR2 overexpression induced endoplasmic reticulum stress and modulated IRE1α/JNK/CHOP signalling

**Fig. 4:**
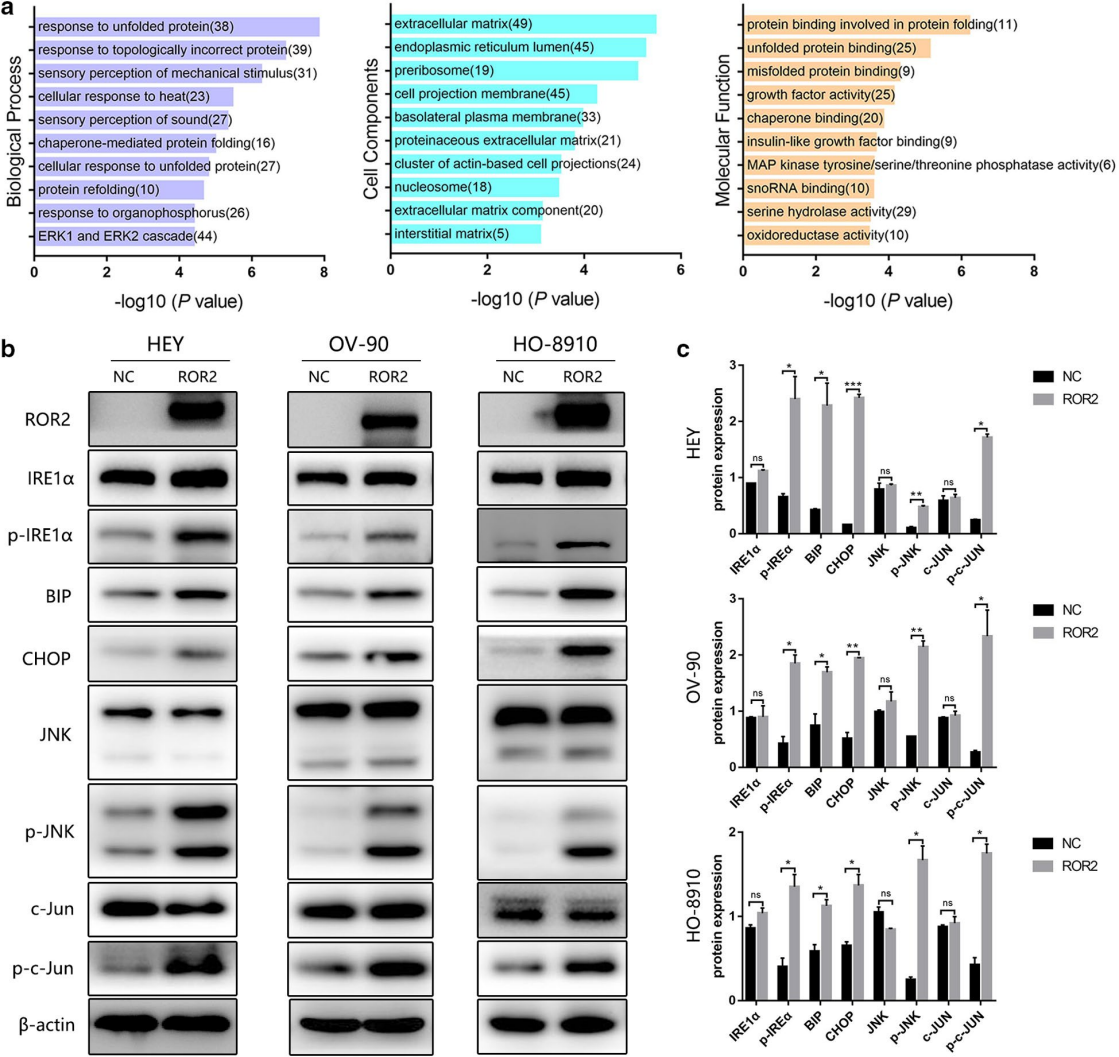
ROR2 overexpression activated IRE1α/CHOP/JNK pathway. **a** Gene ontology analysis was performed in ROR2-overexpressed HO-8910 cells compared to negative control cells. Top ten enriched biological processes, cell components and molecular functions was shown. **b** Cells were transfected with ROR2 overexpression or negative control adenovirus for 72 h. Expression of ROR2, IRE1α, phosphorylated IRE1α, CHOP, JNK, phosphorylated JNK, c-Jun, phosphorylated c-Jun was determined by western-blot assay. β‑actin was used as a loading control. **c** Quantitation of wester-blot assay bands shown in **b** using Image J. Statistical analysis was performed using Student’s *t* test. *NC* negative control, *p-IRE1α* phosphorylated IRE1α (Ser724), *p-JNK* phosphorylated JNK (Thr183/Tyr185), *p-c-Jun* phosphorylated c-Jun (Ser73). All experiments were repeated three times at least. *P < 0.05, **P < 0.01 and ***P < 0.001 for statistical analysis of the indicated groups

To explore how ROR2 induced ovarian cancer cells to apoptosis, whole transcriptome sequencing was used to identify the differentially expressed genes. Compared to the NC group, there were 719 upregulated genes and 1260 downregulated genes in the ROR2 overexpression group (Additional file 2: Figure S2a, b). Gene ontology enrichment analysis of these differential genes showed that the enriched biological processes included response to unfolded protein, response to topologically incorrect protein, chaperone-mediated protein folding, cellular response to unfolded protein, protein refolding. Affected cell components included extracellular matrix, endoplasmic reticulum lumen, cell projection membrane. Impacted molecular function included protein binding involved in protein folding, unfolded protein binding, misfolded protein binding and chaperone binding (Fig. 4a). All the results indicated that changes induced by ROR2 overexpression involved unfolded protein response (UPR). UPR is a mechanism responding to endoplasmic reticulum stress (ERS). Then we detected the expression of ERS relevant proteins in three HGSOC cell lines. As shown in Fig. 4b, ERS related proteins like BIP and phosphorylated IRE1α were upregulated by ROR2 overexpression. Furthermore, the pro-death factors like CHOP, phosphorylated JNK and phosphorylated c-Jun in the ROR2-overexpression group were significantly higher than the NC group (Fig. 4b, c). All the results indicated that ROR2 overexpression could induce ERS and modulate IRE1α/JNK/CHOP signalling, further causing apoptosis.

### IRE1α knockdown reversed the apoptosis and activation of IRE1α/JNK/CHOP pathway induced by ROR2 overexpression

**Fig. 5:**
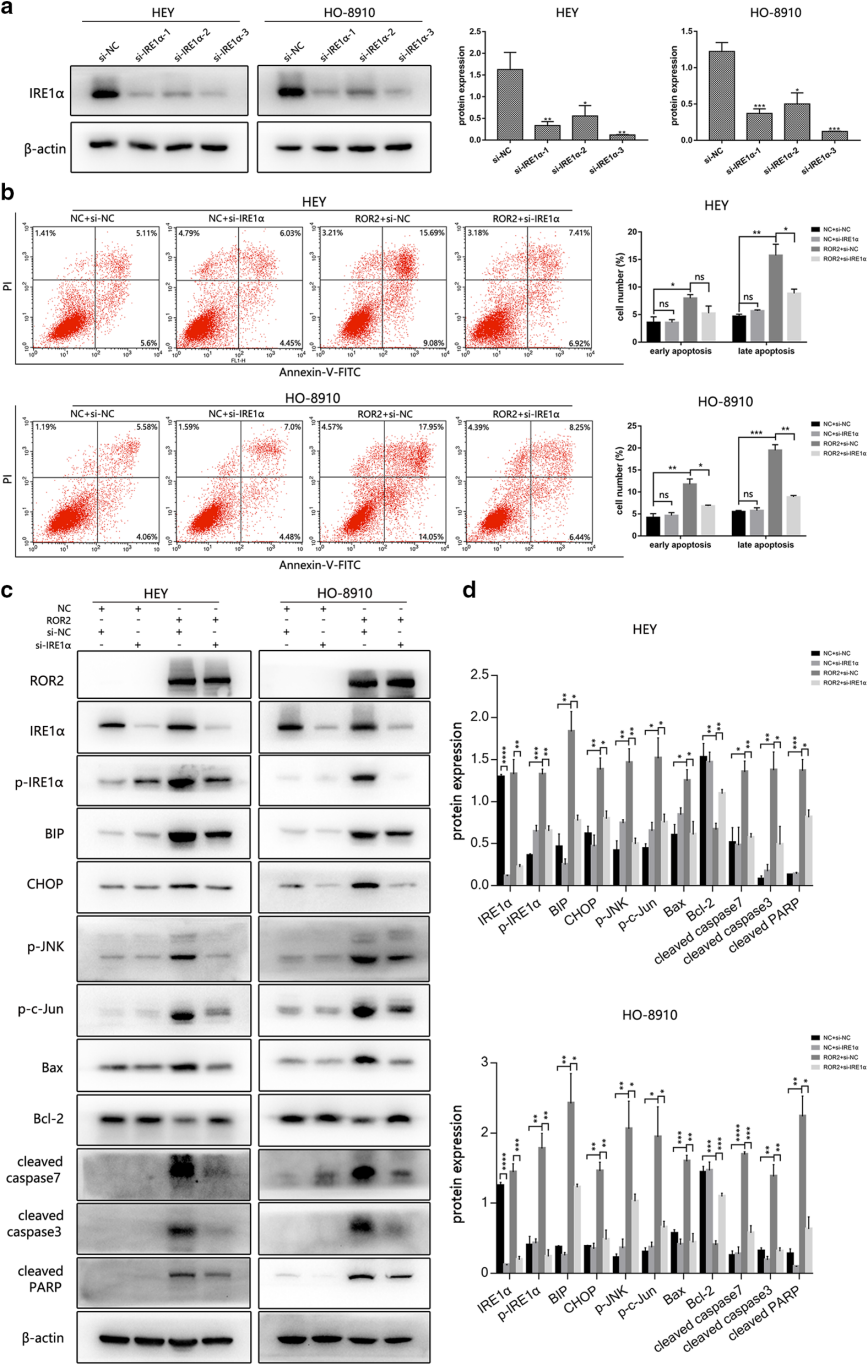
IRE1α knockdown reversed the apoptosis and activation of IRE1α/JNK/CHOP pathway induced by ROR2 overexpression. **a** Expression of IRE1α in HEY and HO-8910 cells were determined by western-blot assay after transfected with si-IER1α or si-NC for 72 h. β‑actin was used as a loading control. **b** HEY and HO-8910 cells were transfected with ROR2 or NC adenovirus for 48 h after pre-transfected with si-IRE1α for 24 h. Apoptosis was detected by flow cytometry after staining with FITC Annexin-V and PI. Quantitive analysis of apoptotic ratio in HEY and HO-8910 cells with CellQuest Pro software. Statistical analysis was performed with GraphPad Prism using Student’s *t* test. **c** HEY and HO-8910 cells were transfected with ROR2 or NC adenovirus for 48 h after pre-transfected with si-IRE1α for 24 h. Expression of ROR2, IRE1α, phosphorylated IRE1α, CHOP, phosphorylated JNK, phosphorylated c-Jun, Bax, Bcl-2, cleaved caspase3, cleaved caspase7, and cleaved PARP were determined by western-blot assay. β‑actin was used as a loading control. **d** Quantitation of western-blot assay bands shown in **c** using Image J. Statistical analysis was performed using Student’s *t* test. *NC* negative control, *p-IRE1α* phosphorylated IRE1α (Ser724), *p-JNK* phosphorylated JNK (Thr183/Tyr185), *p-c-Jun* phosphorylated c-Jun (Ser73). All experiments were repeated three times at least. *P < 0.05, **P < 0.01, ***P < 0.001 and ****P< 0.0001 for statistical analysis of the indicated groups

To further examine whether pro-apoptosis effect of ROR2 was achieved via IRE1α/JNK/CHOP signalling pathway activation, siRNA targeted IRE1α was used to perform the rescue experiment in cells overexpressed ROR2 and corresponding NC cells. The western-blot assay showed nearly 70% of IRE1α expression was suppressed with two sequences (Fig. 5a). We chose the most effective one to do the following experiments. Cells pre-transfected with si-IRE1α or si-NC for 24 h were transfected with ROR2 overexpression or NC adenovirus for following 48 h. Then cells were collected for flow cytometry assay and western-blot assay. We found IRE1α knockdown could alleviate the apoptosis of cells induced by ROR2 overexpression (Fig. 5b). IRE1α knockdown could significantly down-regulate the level of p-IRE1α induced by ROR2 overexpression, therewith strongly inhibited JNK and c-Jun phosphorylation from IRE1α hyperactivation. Moreover, proteins related with apoptosis were detected by western-blot. The change of Bcl-2 and Bax induced by ROR2 were reversed by IRE1α knockdown. IRE1α knockdown could also significantly inhibited caspase3, caspase7 and PARP cleavage upon ROR2 overexpression (Fig. 5c, d).

### Kira6 reversed the apoptosis and activation of IRE1α/JNK pathway induced by ROR2 overexpression

**Fig. 6:**
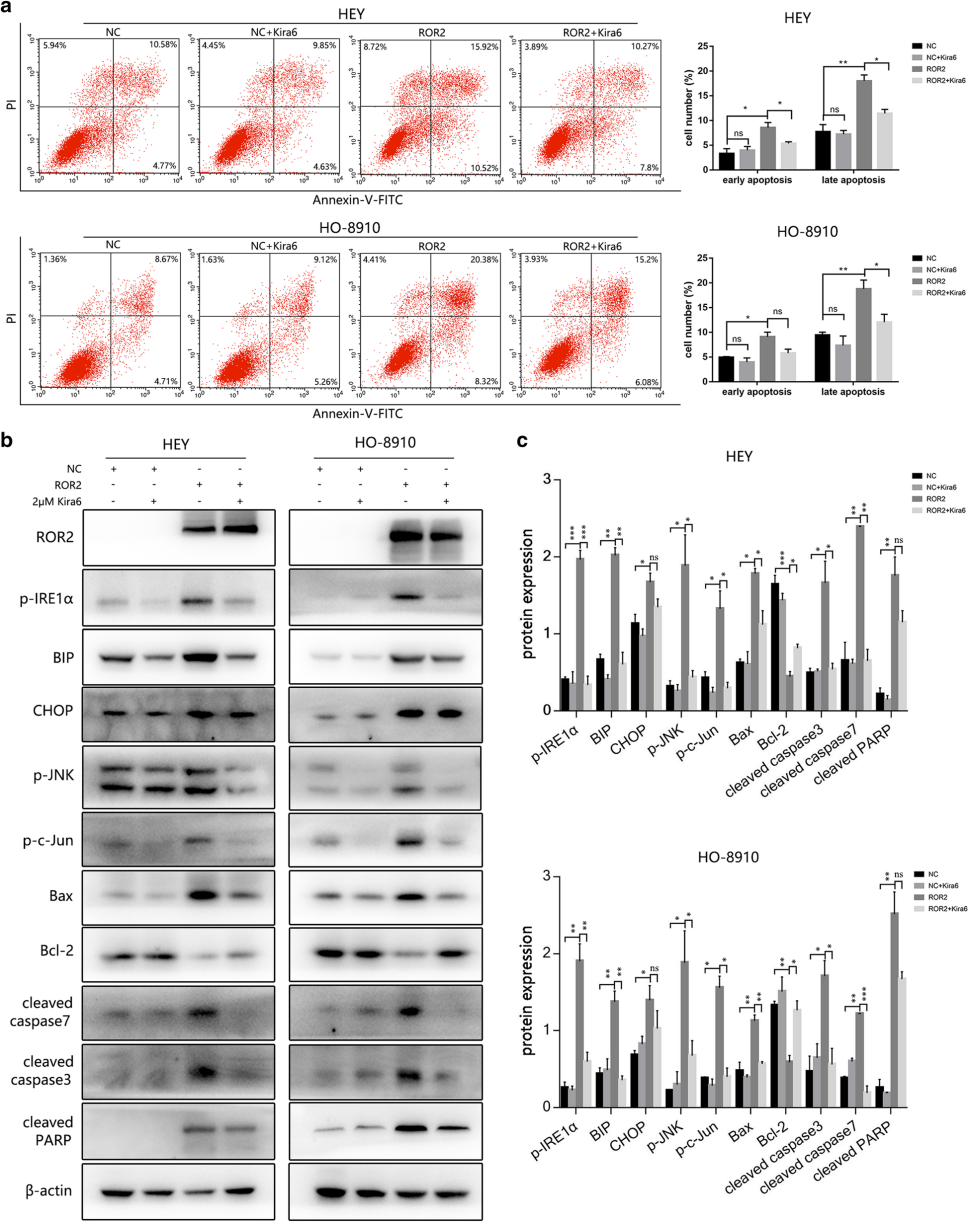
Kira6 reversed the apoptosis and activation of IRE1α/JNK/CHOP pathway induced by ROR2 overexpression. **a** HEY and HO-8910 cells were treated with Kira6 for 72 h after pre-transfected with ROR2 overexpression or negative control adenovirus for 6 h. Apoptosis was detected by flow cytometry after staining with FITC Annexin-V and PI. Quantitive analysis of apoptotic ratio in HEY and HO-8910 cells with CellQuest Pro software. Statistical analysis was performed with GraphPad Prism using Student’s *t* test. **b** HEY and HO-8910 cells were treated with Kira6 for 72 h after pre-transfected with ROR2 overexpression or negative control adenovirus for 6 h. Expression of ROR2, phosphorylated IRE1α, CHOP, phosphorylated JNK, phosphorylated c-Jun, Bax, Bcl-2, cleaved caspase3, cleaved caspase7, and cleaved PARP were determined by western-blot assay. β‑actin was used as a loading control. **c** Quantitation of western-blot assay bands shown in **b** using Image J. Statistical analysis was performed using Student’s *t* test. *p-IRE1α* phosphorylated IRE1α (Ser724), *p-JNK* phosphorylated JNK (Thr183/Tyr185), *p-c-Jun* phosphorylated c-Jun (Ser73). All experiments were repeated three times at least. *P < 0.05, **P < 0.01 and ***P < 0.001 for statistical analysis of the indicated groups

As IRE1α triggers cell apoptosis via its RNase [27], Kira6, an IRE1α’s RNase inhibitor, was used to further verify the above-mentioned results. Cell viability was assayed with the MTT assay after treated with Kria6 for 72 h. Kira6 under 8 μM did not show significant effects on the viability of cells. After pre-transfected with ROR2 overexpression or negative control adenovirus for 6 h, 2 μM Kira6 was added into the culture medium. The cells were subjected to further incubation for 72 h for flow cytometry assay and western-blot assay. Kira6 could alleviate ROR2 induced apoptotic response in HEY and HO-8910 cells as shown by flow cytometry assay (Fig. 6a). The western-blots assay showed Kira6 not only reversed the activation of IRE1α/JNK pathway, but also significantly reversed the change of apoptosis related proteins induced by ROR2 (Fig. 6b, c). However, the upregulation of CHOP couldn’t be significantly reversed by Kira6 treatment. Besides, the inhibition of PARP cleavage by Kira6 was not that significantly.

### ROR2 overexpression suppressed ovarian cancer growth in vivo

**Fig. 7:**
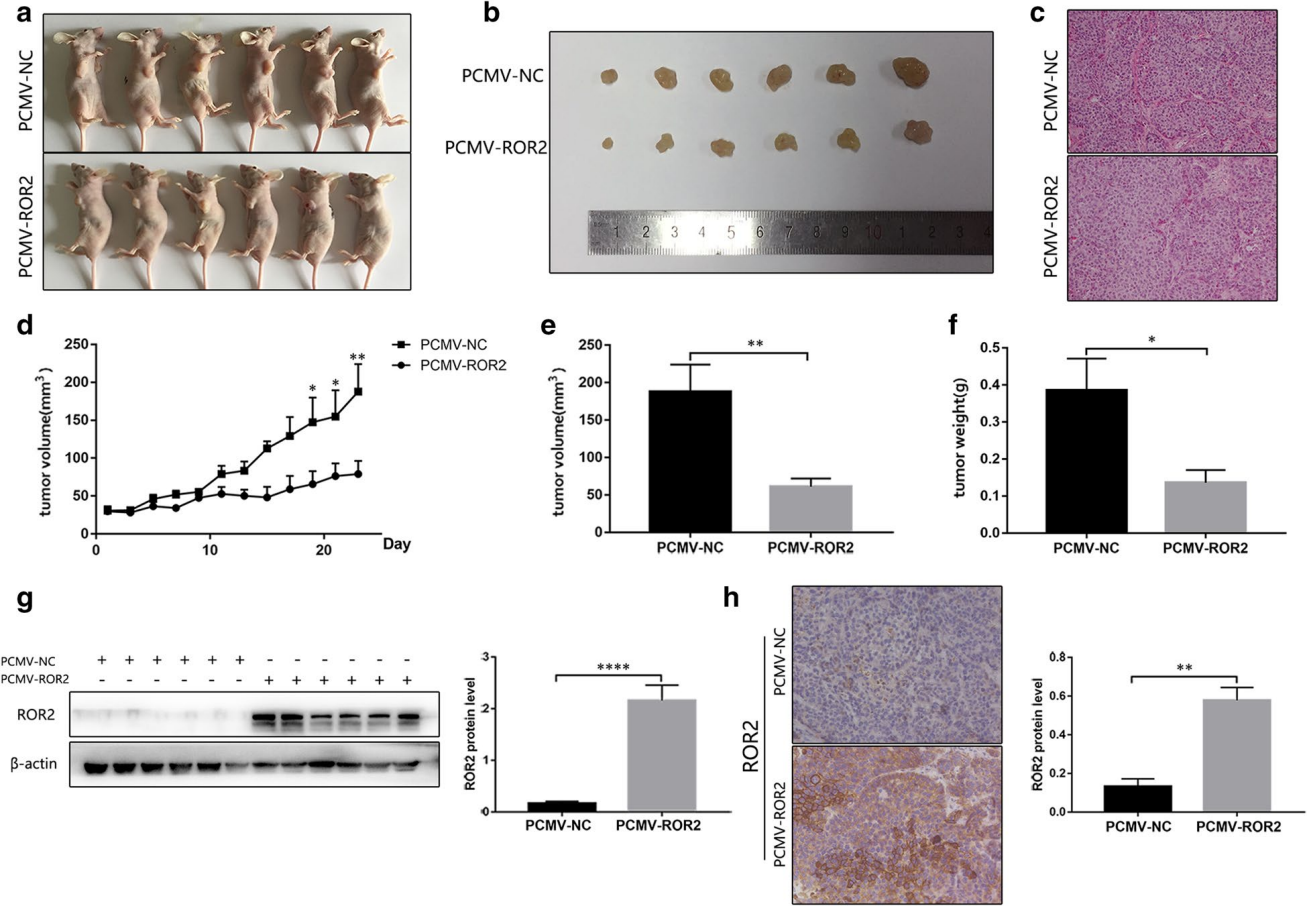
ROR2 overexpression suppressed HGSOC cell growth in vivo. **a** Images of mice with subcutaneous xenograft tumor. **b** Images of tumors transfected with PCMV-ROR2 or PCMV-NC. **c** Images of xenograft tumors’ HE staining. **d** The volumes of tumors with PCMV-ROR2 or PCMV-NC were measured and calculated every other day before the mice were sacrificed. The growth curves were analyzed using 2way ANOVA test. **e**, **f** The volume and weight of the formed tumors transfected with PCMV-ROR2 or PCMV-NC were compared using Student’s *t* test. **g**, **h** Western-blot assay and IHC assay were used to detect the protein levels of ROR2 in the formed tumors. β‑actin was used as a loading control in western-blot assay. The intensities of IHC staining were quantitated by Image-Pro Plus 6.0. IHC scores were analyzed with Student’s *t* test. *P < 0.05, **P < 0.01, ***P < 0.001 and ****P< 0.0001 for statistical analysis of the indicated groups

To further verify the effects of ROR2 on the proliferation assay of ovarian cancer cells, we used HO-8910 cells to construct stable overexpression cell model with Lentivirus PCMV-NC or PCMV-ROR2. 1 × 10^7^ cells in 100 μl PBS were injected subcutaneously into either side of the armpit of the same 4-week-old nude female mice. Tumor sizes were measured every other day from the 10th day after injection. The volume and weight of tumors in the PCMV-ROR2 overexpression group were significantly lower than those in the PCMV-NC group (Fig. 7d–f). Western-blot assay and IHC assay further confirmed the successful overexpression of ROR2 in the ROR2 transfected group (Fig. 7g, h).

## Discussion

ROR2, one of the ROR RTKs, has been brought into focus in cancer field during the past decades. ROR2 was initially found to play critical role in embryo development, like heart, lung, limbs and brain [5, 28, 29]. Mutations of ROR2 could result in human genetic disorders, like Robinow syndrome and brachydactyly type B (BDB) [30–33]. However, the role of ROR2 in human adult tissues has been hardly researched. As the study processed, more and more evidences indicate the complexity of the role of ROR2 in tumor initiation and progression. Upregulation of ROR2 in osteosarcoma, melanoma, renal cell carcinoma, chemoresistant ovarian cancer and the relationship with higher risk diseases defined it as a tumor promoter in early studies [11, 13, 34, 35]. Nevertheless, mass of contrary results brought the tumor promoter definition of ROR2 into controversy. Lara et al. [14] found ROR2 was frequently repressed by promoter hypermethylation in colon cancer cells and tissues and the repression had a tumor-promoting effect. Then Sean et al. [15] corroborated the conclusion. O’Connell et al. [13] reported ROR2-positive melanoma cells had phenotypes of more invasiveness but less proliferation. Li et al. [26] demonstrated ROR2 was frequently methylated in common carcinomas and worked as a tumor suppressor. Ming et al. [16] found ROR2 loss in hepatocellular carcinoma was associated with poor prognosis. Findings above indicated ROR2 played dual roles in tumors depending on the tumor type and tumor context.

In this report, we demonstrated that HGSOC patients with advanced stages were prone to expressed lower ROR2. Restoration of ROR2 in HGSOC cells could repress cell proliferation and induce cell apoptosis. Meanwhile, we also detected the change of invasion and migration abilities in ROR2 overexpression cells. Interestingly, while we found ROR2 overexpression repressed the invasion and migration abilities of EOC cells, the EMT associated protein like N-cadherin, Keratin and Vimentin were hardly changed. Considering the result that ROR2 overexpression could repress EOC cell viability, we speculated that the repression of invasion and migration was on account of decreased cell viability rather than reversion of EMT.

To clear the underlying mechanism of ROR2 in our study, whole transcriptome analysis was used to identify the differentially expressed genes. Gene ontology enrichment analysis of these differential genes showed that the enriched biological processes included response to unfolded protein, response to topologically incorrect protein, chaperone-mediated protein folding, cellular response to unfolded protein, protein refolding. All the changes converged to a point that ROR2 overexpression could induce unfolded protein response.

Endoplasmic reticulum (ER) is an important organelle where proteins are modified, properly folded and calcium saved [36, 37]. Factors like calcium imbalance, inflammation, oxidative stress and so on induced accumulation of unfolded proteins in ER, consequently activating UPR. UPR is originally a mechanism to relieve endoplasmic reticulum stress [38]. Three sensors, inositol requiring kinase1α (IRE1α), double-strand RNA-dependent protein kinase like ER kinase (PERK) and activating transcription factor (ATF6) are released from the binding protein BIP (also named glucose regulated protein 78kda, GRP78) upon UPR [37]. BIP, as the major ER-resident chaperone, is widely used as an ERS biomarker. Adaptive UPR is required for endoplasmic reticulum homeostasis as it could enhance ER capacity and degrade unfolded proteins while uncontrolled and excessive ERS triggers programmed cell death [38]. These three upstream sensors participate in processes of both adaption and destruction, demonstrating other factors are required to determine cell fate.

Several downstream effectors are involved in ERS induced apoptosis. One primary pro-apoptotic protein responding to both IRE1α and PERK (predominantly PERK) signalling pathway is C/EBP homologous protein (CHOP, also known as growth arrest and DNA damage153, GADD153 and DNA-damage inducible transcript 3, DDIT3) [39, 40]. CHOP is barely expressed in normal conditions. During cell stress, like persistent ERS, CHOP is revealed to be a pro-apoptotic transcription factor and plays vital role in ERS induced cell apoptosis [41, 42]. Mechanism underlying pro-apoptotic activity of CHOP includes binding to and regulating genes functioning in cell death, such as Bcl-2, Bim, Bax and Bad [43–45]. Another component involved in ERS mediated apoptosis is c-Jun N-terminal Kinase (JNK) and its canonical target c-Jun [46, 47]. ERS can lead to JNK phosphorylation which in turn activates c-Jun primarily via the IRE1α arm [48]. Sustained activated JNK-c-Jun signalling give rise to pro-apoptotic protein Bax activation and pro-survival factor Bcl-2 inactivation, and induce cell apoptosis [49]. In addition to interaction with Bcl-2 family, other effectors also participate in the ERS mediated apoptosis. Caspase cascades like caspase12, caspase9, caspase3 are also considered as important effectors in response to CHOP/JNK activation [50]. In our study, cells transfected with ROR2 adenovirus for 48 h were used for whole transcriptome analysis, and the results converged to the activation of UPR. Considering the dual roles of UPR in cell fate determination, we used western-blot assay to detect ERS associated proteins in cells transfected with ROR2 for 72 h. Results showed that ROR2 overexpression could upregulate phosphorylated IER1α and BIP, demonstrating the activation of UPR, verifying the RNA sequencing results. Meanwhile, we also detected the expression of CHOP, phosphorylated JNK and phosphorylated c-Jun, and the upregulation of these factors confirmed the ERS mediated cell apoptosis, which was consistent with the flow cytometry assay results and expression changes of Bcl-2 family and caspase cascade. Here we further confirmed that ROR2 overexpression induced cell apoptosis accompanied with activation of IRE1α/JNK/CHOP signalling pathway were reversed by IRE1α knockdown. Meanwhile, rescue experiment was also successfully performed with Kira6, a novel IRE1α type II kinase inhibitor [27]. However, the upregulation of CHOP couldn’t be significantly reversed by Kira6 treatment as CHOP was mainly regulated by PERK [27]. This might explain why Kira6 could only partially reversed the apoptosis, meanwhile indicated CHOP was another molecule leading to the ROR2 induced apoptosis. Along with development of researches, other CHOP/JNK mediated downstream effectors are newly recognized. Han et al. [42] thought instead of directly inducing cell apoptosis, CHOP mediated cell death via increasing protein synthesis and causing ATP depletion and oxidative stress. Li et al. [51] found CHOP could activate calcium-mediated apoptosis in macrophage via inducing transcription of Ero1α. Xu et al. [52] concluded CHOP mediated cell death was achieved via directly regulation of miR-216b. Although ERS and its downstream cascades still require more research, the CHOP/JNK modulation induced cell apoptosis is already well-known.

Limitation of our research is that we didn’t ascertain the bridge between ROR2 and ERS. In regard to the upstream executor of ERS, nutrient deficiency, enhancing Ca^2+^ influx, abnormal protein synthesis and so on are included. Considering that ER is a large reservoir of sequestered Ca^2+^ which plays a crucial role in correct protein folding and modification, loss of ER Ca^2+^ homeostasis inducing ERS is brought into focus. Mechanism how excessive Ca^2+^ release from ER is still poorly understood. In resting state, the concentration of Ca^2+^ in the endoplasmic reticulum was higher than that in the cytoplasm. Ryanodine receptor (RyR), inositol-1,4,5-triphosphatereceptor (IP3R) and Ca^2+^ pump are three channels controlling the release and intake of Ca^2+^ in ER. Thereinto, IP3R is the major intercellular Ca^2+^ release channel. Binding of IP3 initiates IP3R activation. As the concentration of IP3 increases, IP3 drives IP3R clusters formation and eventually allows recruitment of Ca^2+^ release, manifesting a way to induce Ca^2+^ imbalance [53]. Chemicals like endothelin-1 and docosahexaenoic acid (DHA) have been reported to induce ERS via IP3-IP3R interaction [54–56]. Although there is no direct evidence testifying the bond of ROR2 and IP3-IP3R signalling, ROR2 involved Wnt/Ca^2+^ signaling pathway has been confirmed to lead to the production of IP3 and causing the Ca^2+^ release from ER [57]. So, we speculate that ROR2 overexpression actives Wnt/Ca^2+^ signalling pathway, upregulates IP3, releases Ca^2+^ from ER, induces ERS and eventually results in cell apoptosis.

## Conclusions

We found ROR2 was downregulated in HGSOC tissues and its expression was correlated with FIGO stages. Overexpression of ROR2 in HGSOC cells repressed cell proliferation and induced cell apoptosis. We also found ROR2 overexpression could activate IRE1α/JNK/CHOP pathway, which might be the underlying mechanism how ROR2 induced cell apoptosis. Further studies on the relevance of ROR2 and ERS are urgently required to better understand the mechanism of ROR2. All our findings revealed a novel character ROR2 played in HGSOC.

## Supplementary information

**Additional file 1: Figure S1.** ROR2 overexpression inhibited invasion and migration of HGSOC cells. A. Images of HEY, OV-90 and HO-8910 cells invading or migrating through the collagen membrane (×100 magnification). B. Quantification of HEY, OV-90 and HO-8910 cells invading or migrating through the collagen membrane. Statistical analysis was performed using Student’s *t* test. C. Markers associated with EMT in HEY, OV-90 and HO-8910 cells, respectively. β‑actin was used as a loading control. *P < 0.05, **P < 0.01, ***P < 0.001 and ****P<0.0001 for statistical analysis of the indicated groups.

**Additional file 2: Figure S2.** Differentially expressed genes in ROR2-overexpressed HO-8910 cells compared to negative control cells. A. Volcano plot of differential expression results (up-regulated genes are in red; down-regulated genes are in green). B. Heatmap of differentially expressed genes.

## Data Availability

Not applicable.

## Abbreviations

ATF: activating transcription factor
BDB: brachydactyly type B
CHOP: C/EBP homologous protein
DDIT3: DNA-damage inducible transcript 3
DHA: docosahexaenoic acid
ER: endoplasmic reticulum
ERS: endoplasmic reticulum stress
FIGO: International Federation of Gynecology and Obstetrics
FITC: fluorescein isothiocyanate
GADD153: growth arrest and DNA damage153
GRP78: glucose regulated protein 78kda
HGSOC: high-grade serous ovarian carcinoma
IHC: immunohistochemistry
IP3R: inositol-1,4,5-triphosphatereceptor
IRE1α: inositol requiring kinase1α
JNK: c-Jun N-terminal Kinase
NC: negative control
PERK: protein kinase like ER kinase
PI: propidium iodide
PMSF: phenylmethanesulfonyl fluoride
RORs: receptor tyrosine kinase-like orphan receptors
RTKs: receptor tyrosine kinases
RyR: Ryanodine receptor
siRNA: small interfering RNA
SEM: standard error of mean
UPR: unfolded protein response

## References

[1] Torre LA, Trabert B, DeSantis CE, Miller KD, Samimi G, Runowicz CD (2018). Ovarian cancer statistics, 2018. CA Cancer J Clin.

[2] Nezhat FR, Apostol R, Nezhat C, Pejovic T (2015). New insights in the pathophysiology of ovarian cancer and implications for screening and prevention. Am J Obstet Gynecol.

[3] Blume-Jensen P, Hunter T (2001). Oncogenic kinase signalling. Nature.

[4] Forrester WC (2002). The Ror receptor tyrosine kinase family. Cell Mol Life Sci.

[5] Yoda A, Oishi I, Minami Y (2003). Expression and function of the Ror-family receptor tyrosine kinases during development: lessons from genetic analyses of nematodes, mice, and humans. J Recept Signal Transduct Res.

[6] Cui B, Zhang S, Chen L, Yu J, Widhopf GF, Fecteau J-F (2013). Targeting ROR1 inhibits epithelial–mesenchymal transition and metastasis. Cancer Res.

[7] Zhang H, Qiu J, Ye C, Yang D, Gao L, Su Y (2014). ROR1 expression correlated with poor clinical outcome in human ovarian cancer. Sci Rep.

[8] Zhang S, Cui B, Lai H, Liu G, Ghia EM, Widhopf GF (2014). Ovarian cancer stem cells express ROR1, which can be targeted for anti-cancer-stem-cell therapy. Proc Natl Acad Sci USA.

[9] Liu Y, Yang H, Chen T, Luo Y, Xu Z, Li Y, Yang J (2015). Silencing of receptor tyrosine kinase ROR1 inhibits tumor-cell proliferation via PI3K/AKT/mTOR signaling pathway in lung adenocarcinoma. PLoS ONE.

[10] Jung E-H, Lee H-N, Han G-Y, Kim M-J, Kim C-W (2016). Targeting ROR1 inhibits the self-renewal and invasive ability of glioblastoma stem cells. Cell Biochem Funct.

[11] Wright TM, Rathmell WK (2010). Identification of Ror2 as a hypoxia-inducible factor target in von Hippel-Lindau-associated renal cell carcinoma. J Biol Chem.

[12] Yu J, Chen L, Cui B, Widhopf GF, Shen Z, Wu R (2016). Wnt5a induces ROR1/ROR2 heterooligomerization to enhance leukemia chemotaxis and proliferation. J Clin Invest.

[13] O’Connell MP, Marchbank K, Webster MR, Valiga AA, Kaur A, Vultur A (2013). Hypoxia induces phenotypic plasticity and therapy resistance in melanoma via the tyrosine kinase receptors ROR1 and ROR2. Cancer Discov.

[14] Lara E, Calvanese V, Huidobro C, Fernández AF, Moncada-Pazos A, Obaya AJ (2010). Epigenetic repression of ROR2 has a Wnt-mediated, pro-tumourigenic role in colon cancer. Mol Cancer.

[15] Ma SSQ, Srivastava S, Llamosas E, Hawkins NJ, Hesson LB, Ward RL, Ford CE (2016). ROR2 is epigenetically inactivated in the early stages of colorectal neoplasia and is associated with proliferation and migration. BMC Cancer.

[16] Geng M, Cao Y-C, Chen Y-J, Jiang H, Bi L-Q, Liu X-H (2012). Loss of Wnt5a and Ror2 protein in hepatocellular carcinoma associated with poor prognosis. World J Gastroenterol.

[17] Lee SE, Lim SD, Kang SY, Suh SB, Suh Y-L (2013). Prognostic significance of Ror2 and Wnt5a expression in medulloblastoma. Brain Pathol.

[18] Henry CE, Llamosas E, Daniels B, Coopes A, Tang K, Ford CE (2018). ROR1 and ROR2 play distinct and opposing roles in endometrial cancer. Gynecol Oncol.

[19] Roarty K, Pfefferle AD, Creighton CJ, Perou CM, Rosen JM (2017). Ror2-mediated alternative Wnt signaling regulates cell fate and adhesion during mammary tumor progression. Oncogene.

[20] Green JL, Kuntz SG, Sternberg PW (2008). Ror receptor tyrosine kinases: orphans no more. Trends Cell Biol.

[21] Rasmussen NR, Wright TM, Brooks SA, Hacker KE, Debebe Z, Sendor AB (2013). Receptor tyrosine kinase-like orphan receptor 2 (Ror2) expression creates a poised state of Wnt signaling in renal cancer. J Biol Chem.

[22] Henry C, Quadir A, Hawkins NJ, Jary E, Llamosas E, Kumar D (2015). Expression of the novel Wnt receptor ROR2 is increased in breast cancer and may regulate both β-catenin dependent and independent Wnt signalling. J Cancer Res Clin Oncol.

[23] Saji T, Nishita M, Ogawa H, Doi T, Sakai Y, Maniwa Y, Minami Y (2018). Critical role of the Ror-family of receptor tyrosine kinases in invasion and proliferation of malignant pleural mesothelioma cells. Genes Cells.

[24] Martinez S, Scerbo P, Giordano M, Daulat AM, Lhoumeau A-C, Thomé V (2015). The PTK7 and ROR2 protein receptors interact in the vertebrate WNT/planar cell polarity (PCP) pathway. J Biol Chem.

[25] Brinkmann E-M, Mattes B, Kumar R, Hagemann AIH, Gradl D, Scholpp S (2016). Secreted frizzled-related protein 2 (sFRP2) redirects non-canonical Wnt signaling from Fz7 to Ror2 during vertebrate gastrulation. J Biol Chem.

[26] Li L, Ying J, Tong X, Zhong L, Su X, Xiang T (2014). Epigenetic identification of receptor tyrosine kinase-like orphan receptor 2 as a functional tumor suppressor inhibiting β-catenin and AKT signaling but frequently methylated in common carcinomas. Cell Mol Life Sci.

[27] Ghosh R, Wang L, Wang ES, Perera BGK, Igbaria A, Morita S (2014). Allosteric inhibition of the IRE1α RNase preserves cell viability and function during endoplasmic reticulum stress. Cell.

[28] Al-Shawi R, Ashton SV, Underwood C, Simons JP (2001). Expression of the Ror1 and Ror2 receptor tyrosine kinase genes during mouse development. Dev Genes Evol.

[29] Yamada M, Udagawa J, Matsumoto A, Hashimoto R, Hatta T, Nishita M (2010). Ror2 is required for midgut elongation during mouse development. Dev Dyn.

[30] Afzal AR, Jeffery S (2003). One gene, two phenotypes: ROR2 mutations in autosomal recessive Robinow syndrome and autosomal dominant brachydactyly type B. Hum Mutat.

[31] Afzal AR, Rajab A, Fenske CD, Oldridge M, Elanko N, Ternes-Pereira E (2000). Recessive Robinow syndrome, allelic to dominant brachydactyly type B, is caused by mutation of ROR2. Nat Genet.

[32] Schwarzer W, Witte F, Rajab A, Mundlos S, Stricker S (2009). A gradient of ROR2 protein stability and membrane localization confers brachydactyly type B or Robinow syndrome phenotypes. Hum Mol Genet.

[33] Huang D, Jiang S, Zhang Y, Liu X, Zhang J, He R (2014). A new mutation in the gene ROR2 causes brachydactyly type B1. Gene.

[34] Dai B, Yan T, Zhang A (2017). ROR2 receptor promotes the migration of osteosarcoma cells in response to Wnt5a. Cancer Cell Int.

[35] Henry CE, Llamosas E, Djordjevic A, Hacker NF, Ford CE (2016). Migration and invasion is inhibited by silencing ROR1 and ROR2 in chemoresistant ovarian cancer. Oncogenesis.

[36] Mandl J, Mészáros T, Bánhegyi G, Hunyady L, Csala M (2009). Endoplasmic reticulum: nutrient sensor in physiology and pathology. Trends Endocrinol Metab.

[37] Hiss DC, Gabriels GA (2009). Implications of endoplasmic reticulum stress, the unfolded protein response and apoptosis for molecular cancer therapy. Part I: targeting p53, Mdm2, GADD153/CHOP, GRP78/BiP and heat shock proteins. Expert Opin Drug Discov.

[38] Scheuner D, Kaufman RJ (2008). The unfolded protein response: a pathway that links insulin demand with beta-cell failure and diabetes. Endocr Rev.

[39] Kim I, Xu W, Reed JC (2008). Cell death and endoplasmic reticulum stress: disease relevance and therapeutic opportunities. Nat Rev Drug Discov.

[40] Lerner AG, Upton J-P, Praveen PVK, Ghosh R, Nakagawa Y, Igbaria A (2012). IRE1α induces thioredoxin-interacting protein to activate the NLRP3 inflammasome and promote programmed cell death under irremediable ER stress. Cell Metab.

[41] Li F, Zheng X, Liu Y, Li P, Liu X, Ye F (2016). Different roles of CHOP and JNK in mediating radiation-induced autophagy and apoptosis in breast cancer cells. Radiat Res.

[42] Han J, Back SH, Hur J, Lin Y-H, Gildersleeve R, Shan J (2013). ER-stress-induced transcriptional regulation increases protein synthesis leading to cell death. Nat Cell Biol.

[43] McCullough KD, Martindale JL, Klotz LO, Aw TY, Holbrook NJ (2001). Gadd153 sensitizes cells to endoplasmic reticulum stress by down-regulating Bcl2 and perturbing the cellular redox state. Mol Cell Biol.

[44] Puthalakath H, O’Reilly LA, Gunn P, Lee L, Kelly PN, Huntington ND (2007). ER stress triggers apoptosis by activating BH3-only protein Bim. Cell.

[45] Ghosh AP, Klocke BJ, Ballestas ME, Roth KA (2012). CHOP potentially co-operates with FOXO3a in neuronal cells to regulate PUMA and BIM expression in response to ER stress. PLoS ONE.

[46] Fernandes KA, Harder JM, Fornarola LB, Freeman RS, Clark AF, Pang I-H (2012). JNK2 and JNK3 are major regulators of axonal injury-induced retinal ganglion cell death. Neurobiol Dis.

[47] Munemasa Y, Ohtani-Kaneko R, Kitaoka Y, Kumai T, Kitaoka Y, Hayashi Y (2006). Pro-apoptotic role of c-Jun in NMDA-induced neurotoxicity in the rat retina. J Neurosci Res.

[48] Urano F, Wang X, Bertolotti A, Zhang Y, Chung P, Harding HP, Ron D (2000). Coupling of stress in the ER to activation of JNK protein kinases by transmembrane protein kinase IRE1. Science.

[49] Fernandes KA, Harder JM, Kim J, Libby RT (2013). JUN regulates early transcriptional responses to axonal injury in retinal ganglion cells. Exp Eye Res.

[50] Lakshmanan AP, Thandavarayan RA, Palaniyandi SS, Sari FR, Meilei H, Giridharan VV (2011). Modulation of AT-1R/CHOP-JNK-Caspase12 pathway by olmesartan treatment attenuates ER stress-induced renal apoptosis in streptozotocin-induced diabetic mice. Eur J Pharm Sci.

[51] Li G, Mongillo M, Chin K-T, Harding H, Ron D, Marks AR, Tabas I (2009). Role of ERO1-alpha-mediated stimulation of inositol 1,4,5-triphosphate receptor activity in endoplasmic reticulum stress-induced apoptosis. J Cell Biol.

[52] Xu Z, Bu Y, Chitnis N, Koumenis C, Fuchs SY, Diehl JA (2016). miR-216b regulation of c-Jun mediates GADD153/CHOP-dependent apoptosis. Nat Commun.

[53] Prole DL, Taylor CW (2019). Structure and function of IP3 receptors. Cold Spring Harb Perspect Biol.

[54] Jain A, Olovsson M, Burton GJ, Yung H-W (2012). Endothelin-1 induces endoplasmic reticulum stress by activating the PLC-IP(3) pathway: implications for placental pathophysiology in preeclampsia. Am J Pathol.

[55] Takanezawa Y, Nakamura R, Hamaguchi M, Yamamoto K, Sone Y, Uraguchi S, Kiyono M (2019). Docosahexaenoic acid enhances methylmercury-induced endoplasmic reticulum stress and cell death and eicosapentaenoic acid potentially attenuates these effects in mouse embryonic fibroblasts. Toxicol Lett.

[56] Shin J-I, Jeon Y-J, Lee S, Lee YG, Kim JB, Lee K (2019). G-protein-coupled receptor 120 mediates DHA-induced apoptosis by regulating IP3R, ROS and ER stress levels in cisplatin-resistant cancer cells. Mol Cells.

[57] De A (2011). Wnt/Ca^2+^ signaling pathway: a brief overview. Acta Biochim Biophys Sin.

