# Supplementary figures and images for: ROR2 induces cell apoptosis via activating IRE1α/JNK/CHOP pathway in high-grade serous ovarian carcinoma in vitro and in vivo
Source: J Transl Med. 2019 Dec 26;17:428. doi: 10.1186/s12967-019-02178-x (PMC6933631; doi:10.1186/s12967-019-02178-x)

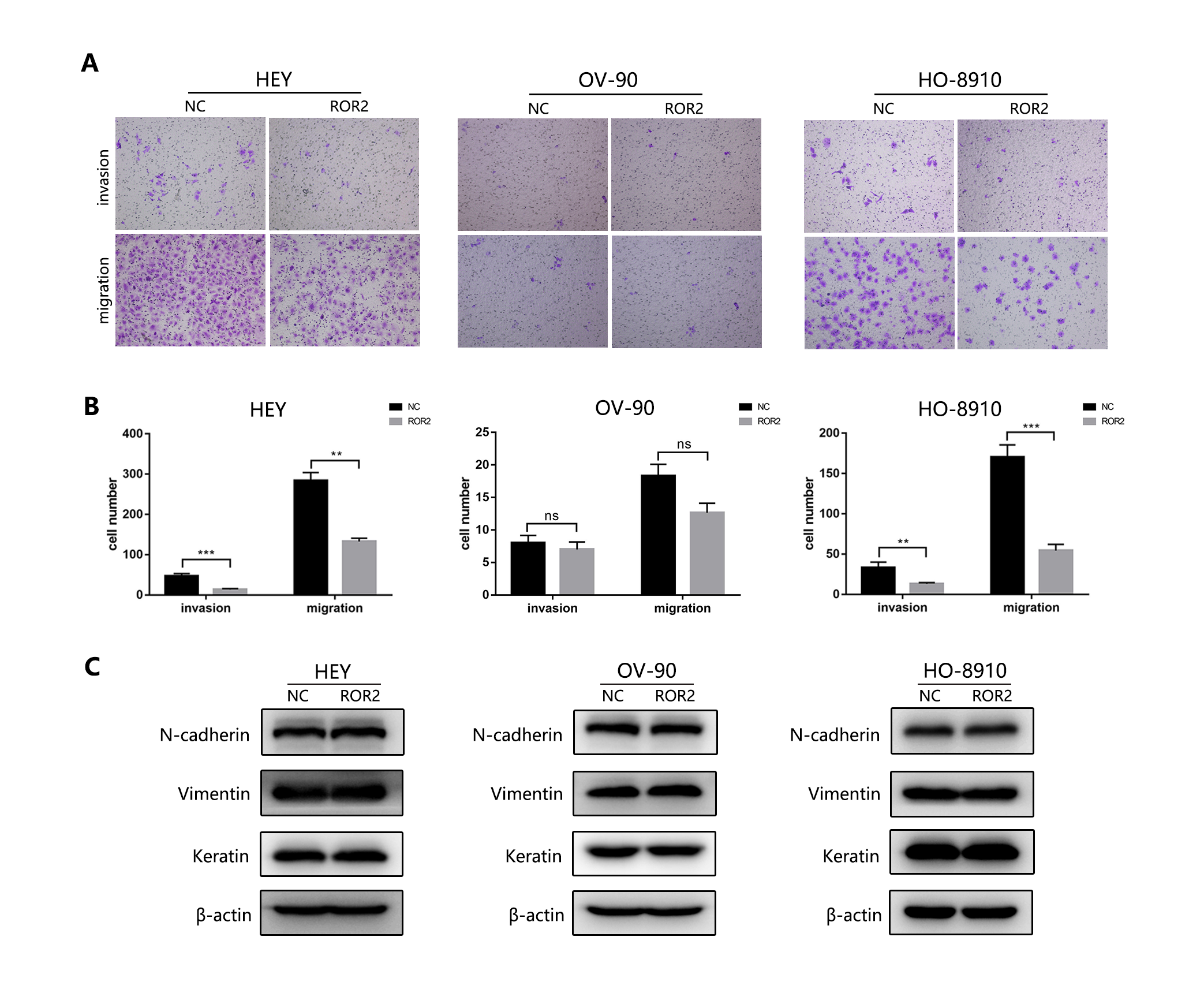

Supplement: Supplementary file 1 — Additional file 1: Figure S1. ROR2 overexpression inhibited invasion and migration of HGSOC cells. A. Images of HEY, OV-90 and HO-8910 cells invading or migrating through the collagen membrane (×100 magnification). B. Quantification of HEY, OV-90 and HO-8910 cells invading or migrating through the collagen membrane. Statistical analysis was performed using Student’s t test. C. Markers associated with EMT in HEY, OV-90 and HO-8910 cells, respectively. β‑actin was used as a loading control. *P < 0.05, **P < 0.01, ***P < 0.001 and ****P<0.0001 for statistical analysis of the indicated groups. [file 12967_2019_2178_MOESM1_ESM.tif]

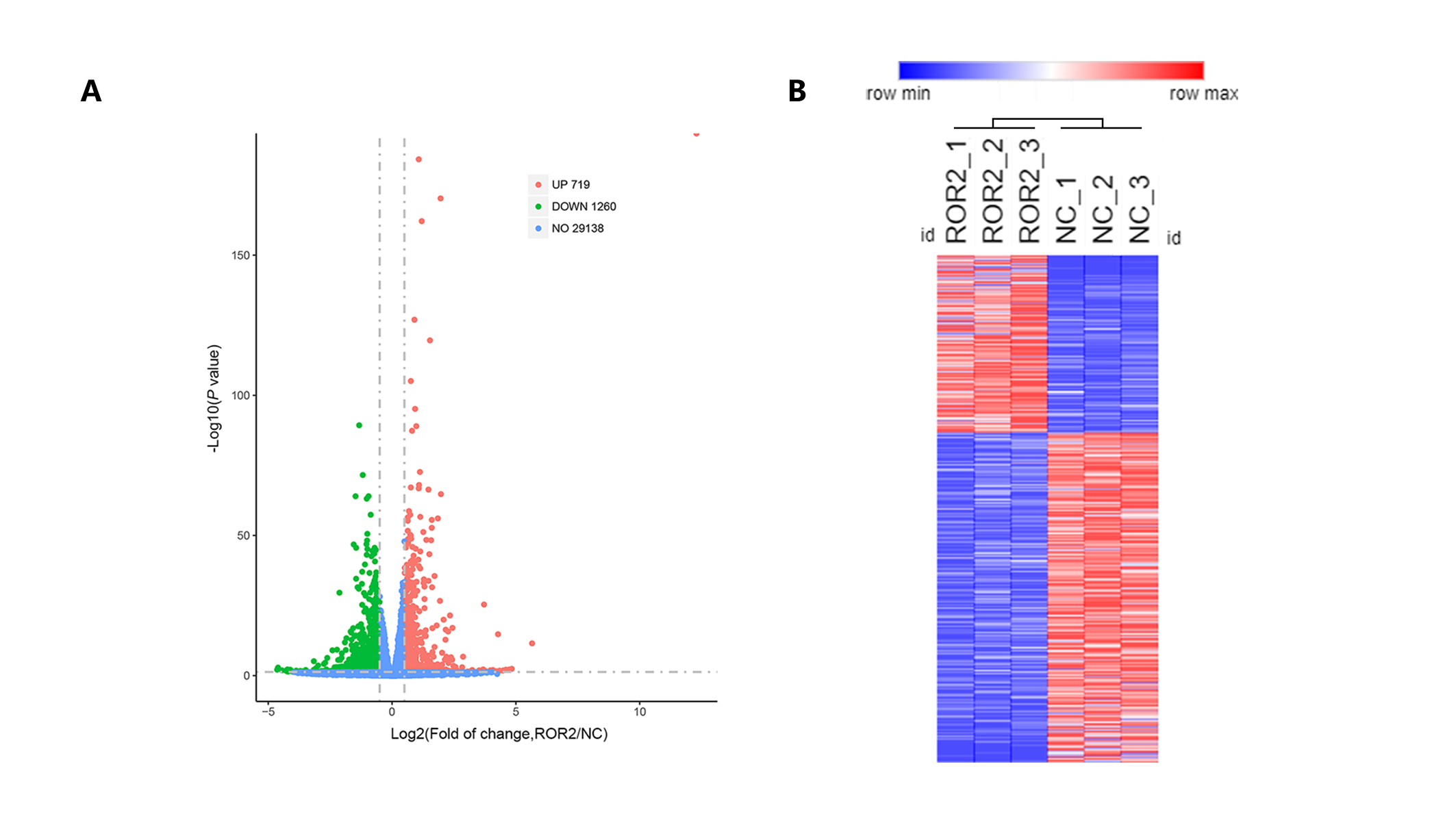

Supplement: Supplementary file 2 — Additional file 2: Figure S2. Differentially expressed genes in ROR2-overexpressed HO-8910 cells compared to negative control cells. A. Volcano plot of differential expression results (up-regulated genes are in red; down-regulated genes are in green). B. Heatmap of differentially expressed genes. [file 12967_2019_2178_MOESM2_ESM.tif]
